# A novel statistical approach for identification of the master regulator transcription factor

**DOI:** 10.1186/s12859-017-1499-x

**Published:** 2017-02-02

**Authors:** Sinjini Sikdar, Susmita Datta

**Affiliations:** Department of Biostatistics, University of Florida, Gainesville, FL 32611 USA

**Keywords:** Master regulator, Transcription factor, Differential connectivity, Regulation, Concordance

## Abstract

**Background:**

Transcription factors are known to play key roles in carcinogenesis and therefore, are gaining popularity as potential therapeutic targets in drug development. A ‘master regulator’ transcription factor often appears to control most of the regulatory activities of the other transcription factors and the associated genes. This ‘master regulator’ transcription factor is at the top of the hierarchy of the transcriptomic regulation. Therefore, it is important to identify and target the master regulator transcription factor for proper understanding of the associated disease process and identifying the best therapeutic option.

**Methods:**

We present a novel two-step computational approach for identification of master regulator transcription factor in a genome. At the first step of our method we test whether there exists any master regulator transcription factor in the system. We evaluate the concordance of two ranked lists of transcription factors using a statistical measure. In case the concordance measure is statistically significant, we conclude that there is a master regulator. At the second step, our method identifies the master regulator transcription factor, if there exists one.

**Results:**

In the simulation scenario, our method performs reasonably well in validating the existence of a master regulator when the number of subjects in each treatment group is reasonably large. In application to two real datasets, our method ensures the existence of master regulators and identifies biologically meaningful master regulators. An R code for implementing our method in a sample test data can be found in http://www.somnathdatta.org/software.

**Conclusion:**

We have developed a screening method of identifying the ‘master regulator’ transcription factor just using only the gene expression data. Understanding the regulatory structure and finding the master regulator help narrowing the search space for identifying biomarkers for complex diseases such as cancer. In addition to identifying the master regulator our method provides an overview of the regulatory structure of the transcription factors which control the global gene expression profiles and consequently the cell functioning.

**Electronic supplementary material:**

The online version of this article (doi:10.1186/s12859-017-1499-x) contains supplementary material, which is available to authorized users.

## Background

Through several scientific findings, it has been suggested that cancer is mainly caused by the mutations in certain genes. So, for effective treatment of cancer, identification of these mutated genes (oncogenes) is very essential. Detailed studies of different cancer datasets often lead to identification of several oncogenes which are directly or indirectly responsible for development and progression of cancer. It is a very challenging task to target and individually study all of these oncogenes as they are large in number. One way to overcome this approach is to group the proteins and genes belonging to the same pathway [1]. These genes and their corresponding pathways are known to form networks that control various cellular functions, and there has been sufficient interest in analyzing such pathway based networks. However recent findings suggest that most oncogenes and tumor suppressor genes encode “transcription factors”, deregulations of which play key roles in carcinogenesis [2, 3]. Majority of the cancer signaling pathways seem to converge to these sets of transcription factors, and these transcription factors lead to tumor development, progression and cancer metastasis through the controlling of the gene expression patterns [2, 3]. As suggested by [2, 4], three main groups of transcription factors, which have been identified for cancer, are the steroid receptors (e.g. estrogen receptors in breast cancer, androgen receptors in prostate cancer), resident nuclear proteins activated by kinase cascades, and the latent cytoplasmic factors (from the STAT protein family members). Apart from these, the ETS protein family members have also been identified as potential cancer transcription factors for their emerging roles in human cancer [5]. It has been shown that direct suppression of these transcription factor expressions can lead to significant antitumor responses with minimal side-effects, and targeting these transcription factors in tumor-related immune cells can help in recovering from tumor immunoresistance [6]. As a result of these features of the transcription factors, in addition to the facts that they are much smaller in number than the oncogenes and have well-regulated expression and activities, transcription factors are gaining popularity as potential therapeutic targets in anti-cancer drug development [3, 4, 7, 8].

In the recent past, many studies have identified a transcription factor or a group of transcription factors as the driving force behind the development of a biological or disease process [9–12]. In order to facilitate such detection there have been attempts to develop statistical methods for accurate identification of transcription factors that regulate large number of genes. To this end most of these methods have been attempted for identification of transcription factors and transcription factor binding sites in cell cycle of yeast and similar organisms using multiple data sources [13–18], while a few of these methods have been applied for human cell as well [19]. In addition, there have been efforts in developing statistical tools for identifying a cluster of transcription factors that cooperatively regulates a large number of genes and the associated disease process [20, 21]. Methods have also been developed for identifying differentially regulated gene sets by integrating regulatory networks of transcription factors and gene expression data [22]. Also, transcription factor activities have been estimated through their effect on target genes [23]. The importance of transcription factor regulation is also evident from the fact that methods have been developed for identifying coordinately activated functional modules from gene expression data. These methods assume that the transcription factor regulated target genes are differentially expressed from non-target genes in the same functional module [24]. In fact there have been several studies for identifying transcription factors under the assumption that co-expression indicates co-regulation [25–27]. The main idea behind such transcription factor regulation is that genes regulated by such transcription factors should have, on an average, significantly different expression levels during one or more cell cycle phases [28]. Besides, there have been studies for identifying groups of important transcription factors through integration of different genomic and epigenomic features [29] and integration of transcriptional and protein interaction networks [30]. Most of these recent methods, including that of [31] and [32], have been directed towards identification of a group of candidate driver transcription factors. Despite the fact that in most cases there are a group of transcription factors that regulate the oncogenes and hence the disease process, it has been seen that there is a hierarchical structure in the regulatory activities of these transcription factors where a ‘master regulator’ transcription factor often appears to control most of the regulatory activities of the other transcription factors and the associated genes [33–35]. According to the definition provided in [34] the “master regulator” transcription factor is at the top of a regulatory hierarchy and must not be under the regulatory influence of any other gene or transcription factor. We use this definition and attempt to finding the “master regulator” transcription factor. This master regulator transcription factor can be targeted for proper understanding of the associated disease process and can be used as a biomarker.

In current literature there is a lack of appropriate statistical methods which use a single data source for accurate identification of such a master regulator among a set of identified transcription factors. In this article, we develop a novel two-step statistical approach to test for the existence of a master regulator transcription factor and for subsequent identification of the master regulator, if it exists, from gene expression data alone. The rest of the article is organized as follows: In the Methods section, we develop our test statistic and describe its underlying motivations for identifying the master regulator transcription factor. In the Results section, we describe a set of simulation experiments to evaluate the performance of our method and also apply our proposed method to two real datasets. Finally, we conclude with a discussion on the utility of our proposed method.

## Methods

We first discuss the biological considerations that motivated the development of our test statistic. We then provide the methodology to formulate the test statistic and use it for the identification of the regulatory circuit of the transcription factors and genes. Finally, we identify the master transcription regulator at the top of the regulation hierarchy.

### Biological considerations

**Fig. 1:**
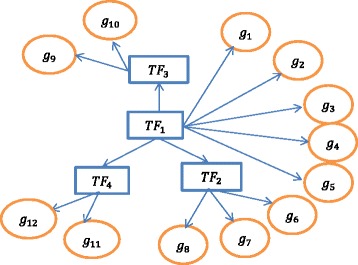
A toy example showing possible regulatory network across a set of genes and transcription factors

Important biological processes can have multiple layers of regulation and control. A transcription factor is known to control not only genes but also other transcription factors. As discussed before in the Background section, usually there is a hierarchical structure in the regulation of the transcription factors so that the master regulator controls most of the regulatory activities of the other transcription factors and the associated genes. In this article, we aim to identify the master regulator transcription factor which is at the top of the hierarchy for better understanding of the associated disease process. A toy example is shown in Fig. 1 which shows the possible regulatory network across a set of genes and transcription factors in a genome.

In Fig. 1, *TF*_1_, *TF*_2_, *TF*_3_and *TF*_4_ denote the transcription factors and *g*_1_, *g*_2_, …, *g*_12_ denote the set of genes. Suppose *TF*_1_ directly regulates five of the genes, which are *g*_1_, *g*_2_, *g*_3_, *g*_4_ and *g*_5_, and also all the other three transcription factors, *TF*_2_, *TF*_3_ and *TF*_4_. The transcription factor *TF*_2_ regulates the genes *g*_6_, *g*_7_ and *g*_8_. Similarly, the transcription factor *TF*_3_ regulates the genes *g*_9_ and *g*_10_ and finally, the transcription factor *TF*_4_ regulates the genes *g*_11_ and *g*_12_. In this example, there exists a hierarchical structure with three layers. We have *TF*_1_ at the top of the hierarchical structure as it directly or indirectly regulates the other transcription factors and the genes. So, *TF*_1_ is considered to be the first layer of the hierarchy. Now, *TF*_1_ directly regulates the other transcription factors, *TF*_2_, *TF*_3_ and *TF*_4_ and the genes *g*_1_, *g*_2_, …, *g*_5_. So, *TF*_2_, *TF*_3_ and *TF*_4_ and *g*_1_, *g*_2_, …, *g*_5_ are considered to be at the second layer of the hierarchy. *TF*_1_ regulates the genes *g*_6_, *g*_7_, …, *g*_12_ indirectly through the transcription factors *TF*_2_, *TF*_3_ and *TF*_4_. Thus, the genes *g*_6_, *g*_7_, …, *g*_12_ form the third layer of the hierarchy. In this example, *TF*_1_ directly or indirectly regulates all the layers of the hierarchy and is not under the regulatory influence of any other gene or transcription factor. Therefore, according to the definition, *TF*_1_ can be considered as the master regulator transcription factor.

Here, in this article, we attempt to develop a test that can check if there exists any transcription factor that acts as a master regulator in a genome, and identify such a master regulator if present. The details of our proposed method are given in the next section.

### Identification of the Master Regulator through a Hypothesis Testing Framework

Let *M* denote the total number of transcription factors present in a genome. Let the transcription factors be denoted by *TF*_1_, *TF*_2_, …, *TF*_*M*_. Let the genes, which are not transcription factors, be denoted by *g*_1_, *g*_2_, …, *g*_*N*_, where *N* denotes the total number of such genes. So, in total, we have expression data on *M* + *N* genes. Let us assume that there are two groups of subjects, for example, the case group (the disease group) and the control group (non-disease group). Let there be *r*_1_ subjects in the case group and *r*_2_ subjects in the control group. So, in other words, we have two groups of subjects with expression levels for *M* + *N* features in each group. It is well known that the genes including the transcription factors are expressed differently in the two groups. Additionally, they are connected with one another differently in the networks of the two groups. There are methods to reverse engineer the networks of genes with association measures such as correlations, partial correlations and partial least squares regression scores [36, 37]. These inter genomic connectivity are different in two groups and can be detected by statistical methods such as Differential Network Analysis (DNA) [38].

Since, it is believed that the master regulator maximally controls the other transcription factors as well as the associated genes; it is important to find the regulatory network among the transcription factors and also the degree of regulation of all the transcription factors on the genes. We first measure the degree of regulation of the transcription factors on the genes. The degree of regulation of a transcription factor on the genes is measured by the change in connectivity of the genes it regulates in the two networks. In other words, we find how the connectivity of a transcription factor with the genes differs between the subjects in case and control groups. For this, we estimate the change in connectivity of a transcription factor with the genes in the two groups of samples using connectivity scores of the given transcription factor with all the genes in the case group with that in the control group. The difference in connectivity is measured using the following statistic [38]:

1 $$ {x}_i=\frac{1}{N}{\displaystyle \sum_{g\in \mathcal{G}}}\left|\ {s}_{T{ F}_i, g}^{case}-{s}_{T{ F}_i, g}^{ctl}\right|\kern2.75em ;\kern1em  i=1,\ 2,\dots, M, $$

where $\mathcal{G}$ denotes the set of all the genes, i.e., the cardinality of $\mathcal{G}$ is *N*. Here, ${s}_{T{ F}_i, g}^{case}\mathrm{and}\ {s}_{T{ F}_i, g}^{ctl}$ are the connectivity scores between the transcription factor *TF*_*i*_, *i* = 1, 2, …, *M*, and the gene *g* in the case and control groups, respectively. There are several choices for connectivity scores such as Pearson’s correlation scores, partial correlation scores, partial least square based association scores. In this article, we use the Pearson’s correlation scores as connectivity scores. So, here, the *x*_*i*_′*s* give us an idea about the magnitude of the differential regulation of the transcription factors on the genes between the case and control groups.

Next, we find the regulatory structure among the transcription factors. For this, we measure the association between each pair of transcription factors using the Pearson’s correlation coefficient scores between them. For each pair (*j*, *k*), let *y*_*jk*_ denote the absolute value of the Pearson’s correlation coefficient score between the transcription factors *TF*_*j*_ and *TF*_*k*_; *j*, *k* = 1, 2, …, *M*, where *y*_*jj*_ = 1. Note that this calculation is done by pooling the data from both the groups.

At this stage, for a transcription factor *TF*_*j*_ , *j* = 1, 2, …, *M*, we have two measures: a measure of the differential regulation of *TF*_*j*_ on the genes (given by *x*_*j*_); and a measure of the association of *TF*_*j*_ with all the transcription factors (*y*_*jk*_,  *k* = 1, 2, …, *M*).

We argue that the degree of change in connectivity of the genes in the two networks is controlled by the transcription factors which are correlated amongst themselves in a hierarchical manner. That is, the hierarchical regulation pattern (as measured by the rank order) among the *M* transcription factors is the same with the differential connectivity of genes in the two groups that they control. In other words, the rank order of the amount of differential connectivity of a transcription factor with other genes it controls is in line (e.g., *concordant*) with its ordered connectivity with the master regulator. Therefore, we consider two ranked lists. One that ranks the transcription factors by the amount of differential connectivity of the genes it controls and another that puts the master regulator in the first position and ranks the remaining transcription factors by their correlation with the master regulator. We evaluate the concordance of these two sets of ranks using a statistical measure which is described in next paragraph. Since we do not know a priori the identity of the master regulator/s, we maximize this measure of concordance over the set of all transcription factors in candidacy for playing the role of the master regulator. In case the maximal *concordance* is statistically significant, we conclude that there is a master regulator. In addition, we declare the transcription factor for which this concordance measure is maximal amongst all transcription factors to be the master regulator.

We construct a concordance statistic *K*_*j*_ for each transcription factor *TF*_*j*_ that is in candidacy for the master regulator ; 1 ≤ *j* ≤ *M*, in the following way:

1. We calculate the Kendall’s rank correlation coefficient test statistic given by (2) below based on the pairs of data ( *x*_1_, *y*_*j*1_), ( *x*_2_, *y*_*j*2_), …, ( *x*_*j*_, *y*_*jj*_), …, ( *x*_*M*_, *y*_*jM*_). Note that *x*_*i*_ denotes the average difference in connectivity of transcription factor *TF*_*i*_ between the two groups, and *y*_*ji*_ is the absolute correlation of transcription factor *TF*_*i*_ with transcription factor *TF*_*j*_. This test statistic *K*_*j*_below conveys whether the differential connectivity of the genes with the transcription factor *TF*_*j*_ in the two experimental groups is concordant with the correlations of the transcription factor *TF*_*j*_with all other transcription factors. In other words, *K*_*j*_ measures whether the differential connectivity is concordant with the hierarchical regulation of the transcription factors amongst themselves. The Kendall’s rank correlation coefficient test statistic for the transcription factor *TF*_*j*_ is given as:
  2 $$ {K}_j=\frac{n_{c, j}-{n}_{d, j}}{n_0}, $$
  where, *n*_*c*,*j*_ = number of concordant pairs in the above paired list,
*n*_*d*,*j*_ = number of discordant pairs in the above paired list,
${n}_0 = \frac{M\left( M-1\right)}{2}$ = Total number of such paired observations for *TF*_*j*_.

This statistic can be used to test the null hypothesis that the two sets of ranks produced by differential connectivity *x* and the correlations with *TF*_*j*_ are non concordant versus the alternative hypothesis that they are concordant.

2) We repeat step 1) for all such transcription factors, so that we have a concordance test statistic for each of the transcription factors which is a potential master regulator.

We believe that the master regulator has the maximum measure of concordance, among all the transcription factors. Since we do not know the identity of the master regulator, we maximize the measure of concordance, given by *K*_*j*_, over the set of all transcription factors. So, we define *K* as the maximum of the statistics given in (2) over all the transcription factors *TF*_*j*_ *s* that are in candidacy for the role of the master regulator, i.e.

3 $$ K=\underset{j}{ \max }{K}_j $$

Thus, statistically significant large values of *K* would indicate the existence of a master regulator.

Significance of *K* can be assessed by a bootstrap (resampling) based procedure as the sampling distribution of *K* is not tractable. This will calculate the *p*-value or the observed level of significance of the value of test statistic *K* calculated in (3). We draw *B* bootstrap samples from the original sample each of size *r*_1_ + *r*_2_ and consider the first *r*_1_ samples as the case group and the remaining *r*_2_ samples as the control group. We compute the test statistic value for each bootstrap sample. Let *K*_*b*_ denotes the value of our test statistic for the *b* ^*th*^ bootstrap sample, where 1 ≤ *b* ≤ *B*. In order to estimate the *p*-value, we calculate the proportion of times the test statistic values based on the bootstrap samples exceed the test statistic value obtained from the original sample, i.e.,

4 $$ \mathrm{p}-\mathrm{value}=\frac{{\displaystyle {\sum}_{b=1}^B} I\left({K}_b > K\right)}{B}\kern0.5em , $$

If the *p*-value obtained from (4) is low then the test is significant and we conclude that there exists a master regulator in the system.

In case we conclude that there exists a master regulator the transcription factor *T* is claimed to be the master regulator if it has the maximum value of the statistic given in (2), i.e.

5 $$ T= \arg \underset{j}{ \max }{K}_j $$

We evaluate the performance of our master regulator identification procedure using a simulation experiment in the next section. A sample test data where our method can be implemented is available in the Additional file 1, while an associated R code for its implementation can be found in http://www.somnathdatta.org/software.

## Results

### Simulation

In order to evaluate the performance of our proposed method, we generate synthetic datasets of gene expressions of the case and control groups with the different regulation schemes of the transcription factors. The simulation scheme consists of the following steps:

### Data Generation

We consider *M* transcription factors *TF*_1_, *TF*_2_, …, *TF*_*M*_ and *N* genes *g*_1_, *g*_2_, …, *g*_*N*_, as described before in the Methods section. Also, let there be *r*_1_ subjects in the case group and *r*_2_ subjects in the control group. The gene expression data for the two groups of subjects are generated as given below. Note that, the choices of all the design parameters considered below are given in later sections depending on whether we are simulating under the null or under the alternative.

1. We assume that (log-transformed) expression values for *TF*_1_ follows a normal distribution with mean *μ* and variance 1 i.e. *N*(*μ*, 1) in the case group, and *N*(*ϑ*, 1) in the control group.
  We also generate *M* independent random variables *V*_*i*_ from *N*(0, 1); *i* = 1, 2, …, *M*, that are also independent of *TF*_1_.
2. We want to generate all the transcription factors in such a way that there exists a hierarchical regulatory pattern among them. In other words, we want to generate the remaining *M* − 1 transcription factors in such a way that *Corr*( *TF*_*j*_ , *TF*_*k*_) > *Corr*( *TF*_*j*_ , *TF*_*l*_ ) (*j* = 1, 2, …, *M*; *k*, *l* = *j* + 1, …, *M*; *k* < *l*), where *Corr*(*TF*_*j*_ , *TF*_*k*_) denotes the correlation between the transcription factors *TF*_*j*_ and *TF*_*k*_. One way of achieving this is to simulate the remaining *M* − 1 transcription factors *TF*_*i*_ ; *i* ≠ 1 as follows:
  $$ T{F}_i=\frac{\rho_i T{F}_1+{V}_i}{\sqrt{1+{\rho}_i^2}}\kern1em ;\kern1.75em  i\ne 1 $$
  where, *ρ*_*i*_′*s* are decreasing in *i*, *i* ≠ 1.
  In this case, the correlation structures among all the transcription factors are given by:
  $$ Corr\left(\  T{F}_1, \kern0.5em  T{F}_i\right) = \frac{\rho_i}{\sqrt{1+{\rho}_i^2}}; \kern0.5em  i\ne 1 $$
  and $$ Corr\left(\  T{F}_j, \kern0.5em  T{F}_k\right)=\frac{\rho_j{\rho}_k}{\sqrt{1+{\rho}_j^2}\sqrt{1+{\rho}_k^2}}\kern0.75em ;\kern0.75em  j, k\ne 1; j\ne k. $$
3. The next step is to generate the genes. We assume that each of the transcription factors *TF*_*i*_ ; *i* = 1, 2, …, *M*, regulates *m*_*i*_ genes. Here, *N* = *m*_1_ + *m*_2_ + … + *m*_*M*_. The genes, ${g}_1,\ {g}_2,\dots,\ {g}_{m_1}$, which are directly regulated by *TF*_1_ alone, are generated as given below:
  $$ {g}_j=\left\{\begin{array}{cc}\hfill T{F}_1{\gamma}_1+{\epsilon}_j\hfill & \hfill \mathrm{for}\ \mathrm{case}\ \mathrm{group}\hfill \\ {}\hfill T{F}_1{\gamma}_2+{\epsilon_j}^{\prime}\hfill & \hfill \kern1.25em \mathrm{for}\ \mathrm{control}\ \mathrm{group}\hfill \end{array}\right.\kern1.75em  j=1,\ 2,\dots,\ {m}_1 $$
  where, *ϵ*_*j*_ and *ϵ*_*j*_′ are independent and identically distributed (i.i.d) as *N*(0, 1), and *γ*_1_ and *γ*_2_ are real numbers.
  Here, the correlation between the transcription factor *TF*_1_ and the genes *g*_*k*_ , *k* = 1, 2, …, *m*_1_ is given by
  $$ Corr\left(\  T{F}_1, \kern0.5em {g}_k\right)=\left\{\begin{array}{cc}\hfill \frac{\gamma_1}{\sqrt{1+{\gamma}_1^2}}\hfill & \hfill \mathrm{for}\ \mathrm{case}\ \mathrm{group}\kern0.5em \hfill \\ {}\hfill \frac{\gamma_2}{\sqrt{1+{\gamma}_2^2}}\hfill & \hfill \kern1.25em \mathrm{for}\ \mathrm{control}\ \mathrm{group}\kern0.5em \hfill \end{array}\right.\kern0.5em  k=1,\ 2,\dots,\ {m}_1 $$
  The genes, regulated by the remaining *M* − 1 transcription factors *TF*_*i*_;  *i* ≠ 1, are generated as follows:
  $$ {g}_j=\left\{\begin{array}{cc}\hfill {V}_i{r}_{1 i}+{\epsilon}_j\hfill & \hfill \mathrm{for}\ \mathrm{case}\ \mathrm{group}\hfill \\ {}\hfill {V}_i{r}_{2 i}+{\epsilon_j}^{\prime}\hfill & \hfill \kern1.25em \mathrm{for}\ \mathrm{control}\ \mathrm{group}\hfill \end{array}\right.\kern2em  j={m}_{i-1}+1,\dots,\ {m}_i $$
  where, *ϵ*_*j*_ and *ϵ*_*j*_′ are i.i.d *N*(0, 1) and *r*_1*i*_ and *r*_2*i*_ are real numbers, *i* ≠ 1.
  In this case, the correlation between a transcription factor *TF*_*i*_;  *i* ≠ 1 and the genes regulated by that transcription factor is given by
  $$ Corr\left( T{F}_i,{g}_k\right)=\left\{\begin{array}{cc}\hfill \begin{array}{cc}\hfill \frac{r_{1 i}}{\sqrt{1+{\rho}_i^2}\sqrt{1+{r}_{1 i}^2}}\hfill & \hfill \mathrm{for}\;\mathrm{case}\;\mathrm{group}\hfill \\ {}\hfill \frac{r_{2 i}}{\sqrt{1+{\rho}_i^2}\sqrt{1+{r}_{2 i}^2}}\hfill & \hfill \mathrm{for}\;\mathrm{control}\;\mathrm{group}\hfill \end{array}\hfill & \hfill \kern1.68em  k={m}_{i-1}+1,\dots,\ {m}_i\hfill \end{array}\right. $$
  Also, the correlations between a transcription factor *TF*_*i*_;  *i* ≠ 1 and the genes which are not regulated by that transcription factors are zero i.e. *Corr*( *TF*_*i*_ , *g*_*k*_) = 0 for *k* ≠ *m*_*i* − 1_ + 1, …, *m*_*i*_. Furthermore, *Corr*( *TF*_1_ , *g*_*k*_) = 0 ; *k* ≠ 1, 2, …, *m*_1_.
  We calculate the size and power of our test in the following sections.

### Size of the Test

Recall that, the null hypothesis of interest is that the rank order of the transcription factors based on their differential connectivity with the genes is not statistically concordant with their rank order based on their correlations with the master regulator. So, the null situation can be created by assuming that there exists a hierarchical regulatory pattern among the transcription factors but there is no differential regulation of the genes in the two experimental groups due to the transcription factors. Hence, there is no such master regulator.

In order to follow the null hypothesis in the simulation setup, we assume *ρ*_*i*_ *s* to be decreasing in *i* , *i* = 2, 3, …, *M* and choose *γ*_1_ = *γ*_2_ and *r*_1*i*_ = *r*_2*i*_, *i* = 2, 3, …, *M*. The decreasing nature of *ρ*_*i*_ *s* ensures that there exists a hierarchical regulatory pattern among the transcription factors. *γ*_1_ = *γ*_2_ and *r*_1*i*_ = *r*_2*i*_, *i* = 2, 3, …, *M* ensure that the associations of the transcription factors with the genes remain the same in the two groups i.e. there is no differential connectivities of the transcription factors with the genes between the two groups. We generate *r*_1_ samples for the case group and *r*_2_ samples for the control group using the above described scheme. We calculate the value of our test statistic, denoted by *K*, using Eq. (3) and find its *p*-value as described in the Methods section.

In order to find the size of the test, we use Monte-Carlo method. We repeat the whole process 1000 times and therefore, get 1000 *p*-values using Eq. (4). Let the *p*-value for the *i* ^*th*^ Monte-Carlo iteration be denoted as *p*_*i*_ , *i* = 1, 2, …, 1000. The size for the test is given by:

6 $$ \mathrm{Size}=\frac{{\displaystyle {\sum}_{i=1}^{1000}} I\left({p}_i<0.05\right)}{1000} $$

In particular, we consider the following choices of the parameters for calculating the size of the test:

- *M* = 10 , *N* = 105, *r*_1_ = *r*_2_ = 500, *B* = 500
- *μ* = 50 , *ϑ* = 5
- *m*_1_ = 30, *m*_2_ = *m*_3_ = … = *m*_7_ = 10 , *m*_8_ = *m*_9_ = *m*_10_ = 5
- *ρ* = (*ρ*_2_, …, *ρ*_10_) = (0.95, 0.8, 0.7, 0.6, 0.5, 0.4, 0.3, 0.2, 0.1)
- *γ*_1_ = *γ*_2_ = 0.5
- *r*_12_ = *r*_22_ = 0.45 and *r*_1*i*_ = *r*_2*i*_ = *r*_1(*i* − 1)_ − 0.05 for *i* = 3, …, 10 .

For the above choices of the parameters, the empirical size of the test came out to be 0.032 which is close to the nominal size of 0.05.

### Power of the Test

To calculate the power of our test, we generate a data under the alternative hypothesis *H*_1_. Here the alternative hypothesis is that the rank order of the transcription factors based on their differential connectivity with the genes is concordant with their rank order based on their correlations with the master regulator. So, we generate the data in such a way that *TF*_1_ acts as the master regulator, that is, the connectivity of *TF*_1_ with other transcription factors are most concordant with the differential connectivity of the genes with the transcription factors. We set *γ*_1_ > *γ*_2_ and *r*_1*i*_ > *r*_2*i*_ ;  *i* = 2, 3, …, 10. Here *γ*_1_ > *γ*_2_ ensures that the connectivity (associations) of *TF*_1_ with the genes, regulated by it, are greater in case group than that in the control group. Similarly, *r*_1*i*_ > *r*_2*i*_ ensures that the connectivity of *TF*_*i*_ with the genes, regulated by it, are greater in case group than that in the control group, *i* = 2, 3, …, 10. Also, we assume *ρ*_*i*_ *s* to be decreasing in *i* , *i* = 2, 3, …, *M*, so that there is a hierarchical regulatory structure among the transcription factors, *TF*_1_ being at the top of the hierarchy. We follow the same steps in calculating the *p*-value as we did for size calculation in the previous section. We consider the same choices for *M*, *N*, *r*_1_, *r*_2_, *B*, *μ*, *ϑ*, *ρ* and *m*_*i*_ ; *i* = 1, 2, …, 10 as we consider for size calculation. In particular, we choose *γ*_2_ = 0.5; *r*_12_ = 0.45 and *r*_1*i*_ = *r*_1(*i* − 1)_ − 0.05 for *i* = 3, …, 10. We choose *r*_2*i*_ = (1 − *δ*)*r*_1*i*_ , *i* = 2, 3, …, 10 where 0 ≤ *δ* ≤ 1. These choices of *r*_2*i*_ , *i* = 2, 3, …, 10; ensure that increase in the value of *δ* also increase the difference between *r*_1*i*_ and *r*_2*i*_ , *i* = 2, 3, …, 10. In other words, the differential regulations of the transcription factors on the genes between the two groups increase as *δ* increases.

For the choice of *γ*_1_, we consider the following relation: *γ*_1_ = *γ*_2_ + *δ*(*r*_12_ − *r*_22_), which implies

**Fig. 2:**
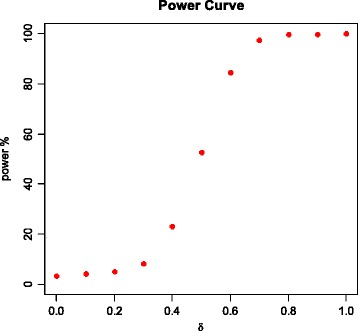
The power curve with 500 subjects in each group for several choices of *δ*

*γ*_1_ = *γ*_2_ + *δ* ^2^ *r*_12_, 0 ≤ *δ* ≤ 1. This choice of *γ*_1_ ensures that increase in the value of *δ* also increase the difference between *γ*_1_ and *γ*_2_. We draw the power curve for different choices of *δ*, as shown in Fig. 2.

From Fig. 2, we see that the power steadily increases as the differential connectivity (regulated by *δ*) of the genes with the transcription factors between the two groups increase. The power curve starts from 3.2% at *δ* = 0 (no difference in the connectivity of the genes with the transcription factors in the two groups) and reaches its maximum of 100% at *δ* = 1 (maximum difference in the connectivity of the genes with the transcription factors in the two groups). The power reaches over 80% with a moderate choice of *δ* = 0.6. Therefore, we can say that our proposed method is a valid test (e.g., size ≤ 0.05) that performs reasonably well (power reaching 100%) in identifying a significant concordance in the differential connectivity of the genes with the transcription factors and the connectivity of a transcription factor with master regulator, if one exists.

We also consider several other choices of the sample sizes in each of the two groups (case and control), and calculate the size and draw the power curves for each of the following choices of the sample sizes: *r*_1_ = 100, *r*_2_ = 70; *r*_1_ = 50, *r*_2_ = 40; and *r*_1_ = *r*_2_ = 50, representing reduced sample sizes and unequal sample sizes in each treatment group. Overall, from our analyses with different choices of sample size, we find that the power of our test is increasing with increase in the sample size as well as an increase in the differential connectivity of the genes with the transcription factors in the two groups. Details of the variation of the power with sample size can be found in Additional file 2 which shows the power curves for each of the above choices of the sample sizes with different choices of *δ*, 0 ≤ *δ* ≤ 1.

In order to check the performance of our test in case there are more than one master regulator transcription factors, we have also studied a simulated scenario where there are two independent master regulator transcription factors regulating two independent sets of genes through transcriptional regulatory networks. Additional file 3 shows the power performance of our test in the presence of two independent master regulators in the system. In this case, too, our test has substantial power performance, similar to the simulated settings of a single master regulator transcription factor. Note that, here we have considered one of the many possible simulation settings. However, our method can also be generalized for several other complicated scenarios.

### Application to Real Datasets

#### Prostate Cancer Data

We apply our test statistic, proposed in Methods section, to a human Illumina expression array dataset GSE18684 of androgen regulated gene expression in the LNCaP prostate cancer cell line [39]. It is believed that androgens and the androgen receptor (AR) play significant roles in prostate cancer cell proliferation and invasion. So, this study was conducted by [39] with an aim to identify the androgen receptor (AR) regulated genes. The LNCaP cells were treated with androgen (R1881) or with vehicle (ethanol) control. There are 10 control and 35 androgen treated samples with expression levels for 17182 probes in the dataset. We identify the set of probes which are differentially expressed in the two groups (androgen treated and vehicle control) using the “limma” package in Bioconductor [40]. After adjusting for false discovery rate (FDR) at 5% significance level, 6054 probes are differentially expressed in the two groups, out of which 542 are transcription factors.

Now, we test whether there exists any master regulator in the above mentioned dataset. For this, we compute the value of our test statistic for this dataset using Eq. (3), which turns out to be 0.49 with a bootstrap based *p*-value of 0.006. Since the *p*-value is highly significant we conclude that there exists a master regulator transcription factor in the system which is controlling all the other transcription factors and the genes. In order to find the master regulator, we use Eq. (5) as given in the Methods section. For this study, the two transcription factors “PEG3” and “ARNT2” have the same value of the test statistic given in Eq. (3). So, we conclude that these two transcription factors maximally control all the other transcription factors and consequently control the connectivity of the genes differently in the two groups. Additionally, the Pearson’s correlation coefficient value between the two transcription factors “PEG3” and “ARNT2” is 0.8. This high value of the correlation suggests that these two transcription factors are approximately at the same level of transcriptional regulatory hierarchy. Therefore, it can be concluded that both of them are the master regulators. Among these two master regulators, “PEG3” has often been linked to the development of prostate cancer. It is believed that deregulation of WNT/ *β* catenin pathway contributes to prostate cancer progression [41–46], and according to [47], inhibition of the transcription factor “PEG3” can lead to enhanced *β* catenin expression and proliferation in human glioma stem cells. This function of the transcription factor “PEG3” is relevant to prostate cancer [48]. Further, the expression of the transcription factor “PEG3” is known to be associated with the processes of cancer aggressiveness and angiogenesis [49]. The results from our analysis show consistency with these known roles of “PEG3” in prostate cancer and demonstrate the utility of our proposed method to identify the master regulator transcription factor. Besides, the transcription factor “ARNT2” is known to have a critical role in human renal tract development, thereby showing congenital abnormalities of the kidneys and urinary tract [50]. “ARNT2” is also known to have significant roles in many cancers like NSCLC [51], breast cancer [52], etc.

Table 1 shows the list of top 10 transcription factors which are highly correlated with the two potential master regulators “PEG3” and “ARNT2”.

**Table 1:** Top 10 transcription factors having high correlations with the master-regulators in the prostate cancer data

Master Regulators	Top 10 transcription factors correlated with the master regulator
PEG3	WWC1, FOXD4L1, NCOA7, TSHZ3, CTBP1, TCFL5, LHX2, ARID5B, CDCA7L, MAK,
ARNT2	MSRB2, TULP4, TSHZ3, TCFL5, SNAPC5, TFDP1, WWC1, CITED4, NCOA7, GRAMD4

From Table 1, it can be seen that the transcription factors “WWC1”, “NCOA7”, “TSHZ3” and “TCFL5” are highly correlated with both the master regulators. Among these, “WWC1” is known to be associated with prostate cancer. The expression of “WWC1” is influenced by AR signaling and is increased in prostate cancer [53]. The transcription factor “NCOA7” is known to affect AR-mediated transcription [54]. The expression of “TSHZ3” is known to be downregulated in prostate cancer [55]. FOXD4L1 is also implicated in many cancers [56].

#### Colorectal Cancer Data

We apply our method to another human microarray dataset GSE4107. This study was conducted by [57] with an aim to identify differentially expressed genes in early onset colorectal cancer (CRC). RNA samples are extracted from colonic mucosa of patients as well as healthy controls and analyzed using GeneChip U133-Plus 2.0 Array. There are 22 subjects involved in the study which included 12 patients and 10 controls. All the patients and the controls in the data are young Chinese who are aged 50 years or less. There are expression levels for 54,675 genes for all the patients in the dataset. We first filter the data in order to find the set of differentially expressed genes between the case and the control groups. For this purpose we use the “limma” package in Bioconductor [40]. After adjusting for FDR at 10% significance level, the number of differentially expressed genes turns out to be 5192, among which 266 are transcription factors.

Next, we apply our method to the filtered dataset. We first test whether there exists a master regulator in the data. The value of our test statistic, given in Eq. (3), is 0.38 for this dataset with a *p*-value of 0.04 for the bootstrap based test. Since, the *p*-value is small enough to make the test significant, we conclude that there exists a master regulator in the data. We identify the master regulator using Eq. (5), given in the Methods section. The master regulator in this data is the transcription factor “NFKB2”. Hence, we conclude that the transcription factor “NFKB2” maximally controls all the transcription factors and the genes in the data.

The transcription factor “NFKB2” is a subunit of the transcription factor nuclear factor-kappa-B (NFKB). “NFKB” transcription factors are known to be the key regulators of innate immune responses, inflammation, and cell survival [58, 59]. Also, “NFKB” activation has been frequently associated with tumor growth in leukemias and lymphomas, as well as prostate, pancreatic and colorectal cancers [60–62]. It has been widely suggested that “NFKB” activation plays a leading role in regulation of target genes that promote cell proliferation, anti-apoptosis, regulate immune and inflammatory response, and results in pathogenesis of various cancers [59, 63–67]. Further, it has been shown that constitutive activation of “NFKB” instigates strong resistance to chemotherapy and radiotherapy [67], while molecular targeted therapy against “NFKB” activation is believed to be effective in colorectal carcinomas with constitutive “NFKB” activation [59]. According to [66], “NFKB” may contribute to the promotion of the ongoing inflammatory process in the gut mucosa resulting in the progression of colitis associated colorectal cancer. Besides, it is believed that “NFKB” activation is involved in development of not only colitis-associated cancer, but also sporadic colorectal cancer [68].

From our data, we find that the master regulator “NFKB2” is maximally (negatively) correlated with the transcription factor “PPARGC1A (PGC-1alpha)” with an overall correlation value of -0.76. The correlation of “NFKB2” and “PPARGC1A” is -0.72 in the patients group whereas it is -0.39 in the control group. It is known that “NFKB” directly repress the activity of “PPARGC1A” in cardiac cells. This leads to the increase in glucose oxidation which is observed during pro-inflammatory state [69].

## Discussion

In this article, we present a novel approach to identify a master regulator transcription factor in a system using only the gene expression profiles of the patients. We consider a simulation setting which validates our approach with a reasonable power in detecting the existence of a master regulator. We have also checked the power of our test in the presence of two independent master regulator transcription factors in the simulation setup. We apply our approach to two human microarray datasets and detect the existence of master regulators in those. In order to check the robustness of our method in experiments not typically falling under the ‘case-control’ category, we have applied our method to an additional dataset, namely, Glioblastoma (GBM) TCGA RNA-seq data [70]. Here we compare the two types of GBM tumors: Mesenchymal and Classical. Our method concludes the existence of a master regulator transcription factor (PPRC1) between the two types of GBM tumors (Mesenchymal and Classical) with a *p*-value of 0.08 (marginally significant).

Our method is aimed to identify a single master regulator, as opposed to identifying a group of transcription factors associated with the disease process as in the case of other existing methods. The method can identify multiple master regulator transcription factors if they are individually at the top of hierarchy of the transcription regulation. This is advantageous in anti-cancer drug development processes which initially target the most potential transcription factor associated with the disease and can be used as a potential biomarker. However, there is a scope of further improvement of our proposed method by incorporating important platforms like ChIP-Seq data. From simulation settings, we see that the performance of our method gets better with the increase in the number of patients in each group. So, our method is expected to be more efficient when there is sufficiently large number (around 100) of patients in each group while it may not be very efficient in case the sample size is very small. Although both the data analyzed in this article have much lower number of subjects in each group, our test was still successful in identifying master-regulator transcription factors from the data. One important assumption of our method is that the ranking of the transcription factors on the basis of their differential connectivity of the genes between two experimental conditions is concordant with the hierarchical order of their own regulation. The fulfilment of the above mentioned condition is a key indicator to the existence of a master regulator transcription factor and its subsequent detection through our method. However, it may be possible that in certain situations, although there exists a master regulator transcription factor, there is no such clear cut concordance between it’s regulation on other transcription factors and differential connectivity with the other genes. In such a case, our method may not perform well.

## Conclusion

We have developed a method of identifying the ‘master regulator’ transcription factor using only the gene expression data. This is advantageous in terms of narrowing down the search space for potential candidate transcription factor biomarkers that can be targeted for drug development of complex diseases. Also, the fact that our method uses only a single data source, e.g. gene expression data, for accurately identifying the master regulator transcription factor makes it very useful in case there is limitation in data sources and data from multiple platforms are not available. In addition to identifying the master regulator our method provides an overview of how the transcription factors regulate the global gene expression profiles and consequently the cell functioning. Additionally, with our method, one can identify many other transcription factors involved in the regulatory roles by reporting the hierarchy amongst them using the rankings of the test statistics values. Overall, we believe that our method will give new insight for efficient identification of potential disease biomarker and therapeutic target in drug development processes.

## Additional files

Additional file 1: Test Dataset. This file contains an example test dataset where our method can be implemented. This simulated data contains 10 transcription factors, namely *TF*_1_, *TF*_2_, …, *TF*_10_ along with 105 genes that were regulated by these transcription factors. Among the transcription factors, *TF*_1_ was generated to play the role of the master regulator. (CSV 1382 kb)
Additional file 2: Figure. Plot of the power curves for different choices of the sample sizes with several choices of *δ*, using simulated datasets. (DOCX 19 kb)
Additional file 3: Figure. Plot showing the power performance of our test in presence of two independent master regulators with varying *δ*, using simulated datasets. (DOCX 16 kb)

## Abbreviations

ChIP-Seq: ChIP Sequencing
FDR: False discovery rate
GBM: Glioblastoma
NSCLC: Non-small cell lung cancer
RNA-Seq: RNA Sequencing
TCGA: The Cancer Genome Atlas
