# Supplementary material for: A novel statistical approach for identification of the master regulator transcription factor
Source: BMC Bioinformatics. 2017 Feb 2;18:79. doi: 10.1186/s12859-017-1499-x (PMC5288875; doi:10.1186/s12859-017-1499-x)
Supplement: Supplementary file 2 [file 12859_2017_1499_MOESM3_ESM.docx]

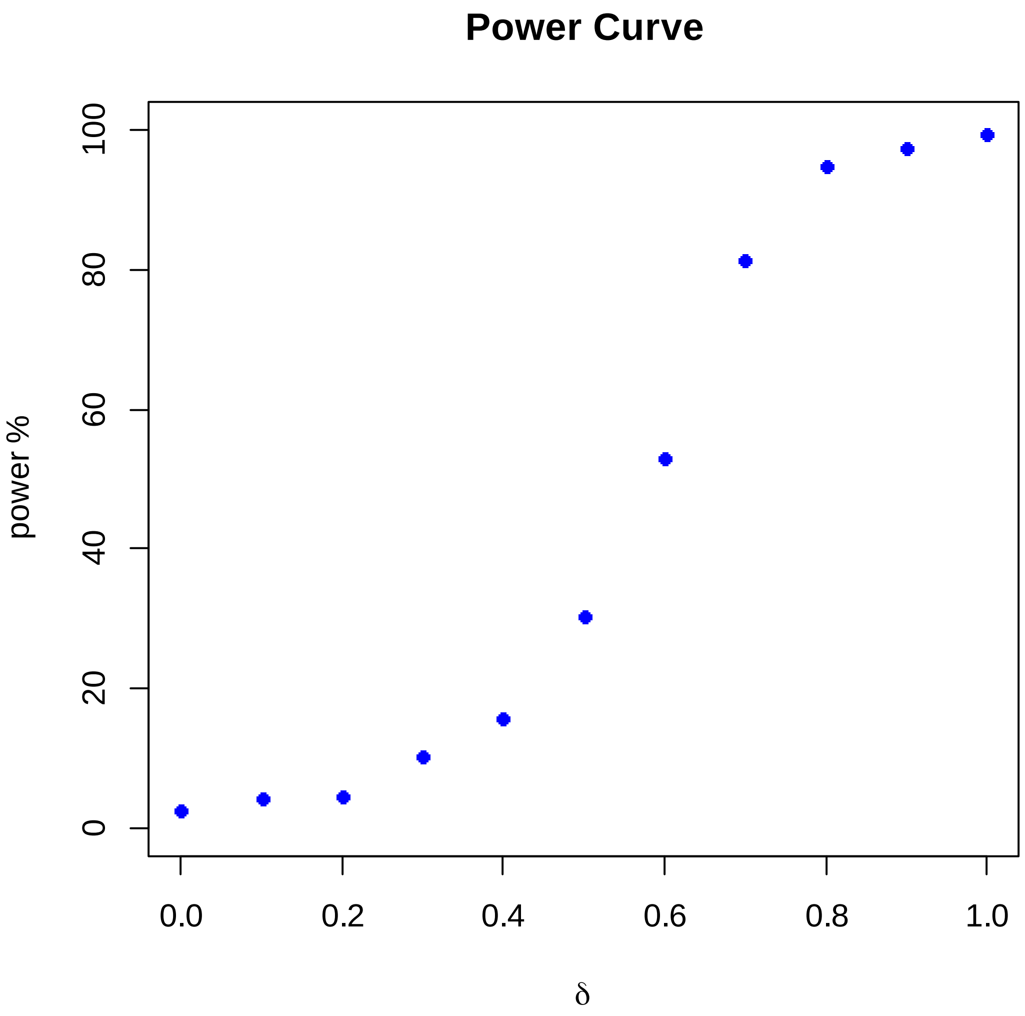
Power Curve
power %
0
20
40
60
80
100
0.0
0.2
0.4
0.6
0.8
1.0
δ
